# Predição do Consumo de Oxigênio de Pico em Pacientes Cardiopatas com Base no Desempenho no Teste *Timed Up and Go*

**DOI:** 10.36660/abc.20230338

**Published:** 2023-12-15

**Authors:** Danilo Silva dos Santos, Ciro Oliveira Queiroz, Cristiane Maria Carvalho Costa Dias, Gerson Cipriano, Queila Oliveira Borges, Luiz Eduardo Fonteles Ritt

**Affiliations:** 1 Escola Bahiana de Medicina e Saúde Pública Unidade Acadêmica Brotas Salvador BA Brasil Escola Bahiana de Medicina e Saúde Pública – Unidade Acadêmica Brotas, Salvador, BA – Brasil; 2 Universidade Estadual do Sudoeste da Bahia Jequie Brasil Universidade Estadual do Sudoeste da Bahia – Campus de Jequie, Jequie – Brasil; 3 Universidade de Brasília Brasília DF Brasil Universidade de Brasília, Brasília, DF – Brasil; 4 Hospital Cardio Pulmonar Centro de Estudos Clínicos Salvador BA Brasil Hospital Cardio Pulmonar – Centro de Estudos Clínicos, Salvador, BA – Brasil

**Keywords:** Consumo de Oxigênio, Doenças Cardiovasculares, Teste de Esforço

## Abstract

**Fundamento:**

A utilização do teste *timed up and go* (TUG) na avaliação da aptidão cardiorrespiratória em cardiopatas não está bem definida na literatura.

**Objetivos:**

Testar a associação entre o TUG e o consumo de oxigênio de pico (VO_2_pico), construir uma equação com base no TUG para prever o VO_2_pico e determinar um ponto de corte para estimar um VO_2_pico ≥ 20 mL.kg−1.min−1.

**Métodos:**

Estudo transversal com 201 indivíduos portadores de doença arterial coronariana ou insuficiência cardíaca, com idade entre 36 e 92 anos, submetidos ao TUG e ao teste cardiopulmonar de exercício. Foram realizadas análises de correlação, curva ROC, regressão linear múltipla e Bland-Altman. Um p < 0,05 foi adotado como significante.

**Resultados:**

A média de idade da amostra total foi 67 ± 13 anos, e 70% dos participantes eram do sexo masculino. A média de VO_2_pico foi de 17 ± 6 mL.kg−1.min−1 e a média de desempenho no TUG foi de 7 ± 2,5 segundos. A correlação entre o VO_2_pico e o TUG foi r = −0,54 (p < 0,001) e R^2^ de 0,30. Foi desenvolvida a equação com base no $\text{ TUG: }{VO}_{2}\text{ pico }=33,553+(-0,149*\text{ idade })+(-0,738*TUG)+(-2,870*\text{ sexo })$ ; sendo atribuído o valor 0 ao sexo masculino e 1 ao sexo feminino (R ajustado: 0,41; R^2^ ajustado: 0,40). O VO_2_pico estimado pela equação foi 18,81 ± 3,2 mL.kg−1.min−1 e o determinado pelo teste cardiopulmonar de exercício foi 18,18 ± 5,9 mL.kg−1.min−1 (p > 0,05). O melhor ponto de corte para o VO_2_pico ≥ 20 mL.kg−1.min−1 foi de ≤ 5,47 segundos (área sob a curva: 0,80; intervalo de confiança de 95%: 0,74 a 0,86).

**Conclusões:**

O TUG e o VO_2_pico apresentaram associação significativa. A equação preditiva do VO_2_pico foi desenvolvida e validada internamente com bom desempenho. O ponto de corte no TUG para prever um VO_2_pico ≥ 20 mL.kg−1.min−1 foi ≤ 5,47 segundos.

## Graphical abstract

**Figura Central:**
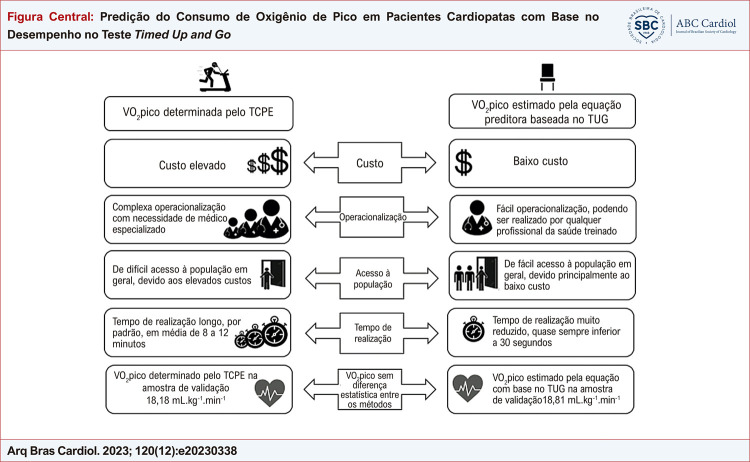
: Predição do Consumo de Oxigênio de Pico em Pacientes Cardiopatas com Base no Desempenho no Teste *Timed Up and Go*

TCPE: teste de esforço cardiopulmonar; TUG: timed up and go; VO_2_pico: consumo de oxigênio no pico do esforço.

## Introdução

As doenças cardiovasculares são as principais causas de morte no mundo, sendo responsáveis por 17,9 milhões dos óbitos no ano de 2019, o que correspondeu a 32% de todas as mortes.^1^ As doenças cardiovasculares são altamente incapacitantes, provocando a diminuição da capacidade funcional (CF), condição que pode sugerir riscos cardiovasculares graves e indica pior prognóstico dos pacientes.^2-5^

A CF é a aptidão para realizar atividades diárias de maneira independente e é considerada um importante indicador de saúde, pois está associada à qualidade de vida.^6^ A CF pode ser avaliada pelo consumo máximo de oxigênio no pico do esforço (VO_2_pico) que é o determinante da aptidão cardiorrespiratória (ACR) da população em geral e valores de VO_2_pico ≥ 20 mL.kg^1^.min^1^ estão relacionados a melhor prognóstico dos avaliados. O teste cardiopulmonar de exercício (TCPE) é o método padrão-ouro para mensurar a ACR, entretanto, não é um teste muito acessível, pois necessita de equipamentos de custo elevado, instalações apropriadas e ser conduzido por um médico especialista, tornando-o um procedimento oneroso e restritivo à maioria da população.^4,7^

Atualmente, testes submáximos validados, como o teste de caminhada de 6 minutos (TC6M) e o teste do degrau de 6 minutos, são alternativas viáveis ao TCPE na avaliação da ACR.^5^É recomendado mensurar periodicamente a CF na população de cardiopatas, pois trata-se de um indicador de prognóstico funcional, clínico e, consequentemente, de mortalidade.^5,7^Portanto, na impossibilidade de realizar o TCPE ou os demais testes funcionais, são empregados outros instrumentos capazes de realizar esta avaliação funcional.

O teste *timed up and go* (TUG) avalia a mobilidade funcional com base na força muscular de membros inferiores, no equilíbrio e na agilidade.^8-10^ É um teste simples e o desempenho considera o tempo em segundos para que o avaliado se levante de uma cadeira e o mais rapidamente, caminhe em linha reta por três metros, vire-se e retorne à cadeira sentando novamente.^11^Dados sobre a utilização e o desempenho no TUG em cardiopatas ainda são escassos.

Sendo assim, o objetivo principal deste estudo foi de construir uma equação preditora do VO_2_pico com base no desempenho do TUG de indivíduos cardiopatas, como também, analisar a associação entre o TUG e o VO_2_pico e determinar no TUG um ponto de corte para definir pacientes com melhor ACR.

## Métodos

Este é um estudo transversal a partir da análise dos dados de participantes de um programa de reabilitação cardíaca, no período de agosto de 2017 a março de 2020, que, obedecendo diretrizes clínicas, realizaram o TCPE e o TUG, em um hospital de referência em cardiologia, na cidade de Salvador, Brasil.

Foram incluídos neste estudo, pacientes acometidos por doença arterial coronariana (DAC) e/ou insuficiência cardíaca (IC), diagnosticadas pela história clínica dos pacientes (infarto agudo do miocárdio, DAC estável, procedimentos de angioplastia ou revascularização ou, ainda, presença de angina ou dispneia), presença de anormalidades eletrocardiográficas ou ecocardiográficas, sendo utilizado o método de Simpson para medida da fração de ejeção. Foram excluídos os participantes que não realizaram o TCPE e o TUG.

Na avaliação inicial, foram coletados os dados clínicos, sociodemográficos e realizado o TCPE. O TCPE foi realizado utilizando uma esteira da marca Micromed (São Paulo), modelo Centurion 300, com um analisador de gases da marca Cortex inc (Leipzig, Alemanha), modelo Metalizer 3b que possui capacidade de mensurar a cada respiração. A classe funcional de cada paciente determinou o protocolo de rampa utilizado, objetivando uma padronização para os testes que tiveram duração entre 8 e 12 minutos. Os dados ventilatórios obtidos foram analisados em intervalos de 10 segundos e o VO_2_pico foi expresso em mL.kg^1^.min^1^. Para a verificação da percepção de esforço, foi utilizada a escala de Borg modificada.

O TUG foi realizado sob supervisão de profissional de saúde treinado, em um intervalo de 2 a 7 dias após a realização do TCPE. No TUG foi utilizado uma cadeira com assento a 46 cm de altura do solo, com encosto para as costas e sem apoio para os braços. Na posição inicial, o avaliado encontrava-se sentado na cadeira, recostado e com os pés apoiados no chão. Para realização do TUG, os participantes foram orientados que ao comando de “levante e vá”, momento em que o cronômetro era acionado, deveriam se levantar sem auxílio dos braços, caminhar o mais rapidamente possível e, ao cruzar uma linha posicionada a 3 metros de distância da cadeira, dar meia volta e retornar para a cadeira, sentando novamente, momento este em que o cronômetro era travado. O desempenho no teste TUG correspondeu ao tempo em segundos necessários para realização deste processo, determinado pelo cronômetro que era administrado por um avaliador treinado para o protocolo.

O protocolo do estudo foi submetido ao Comitê de Ética em Pesquisa Celso Figueirôa no Hospital Santa Izabel e foi aprovado sob o número do CAAE 57813016.0.3001.5533, respeitando as Diretrizes de Helsinque para a realização de pesquisas clínicas e a resolução 466/12 do Conselho Nacional de Saúde. Todos os participantes do estudo assinaram o Termo de Consentimento Livre e Esclarecido.

## Análise estatística

A determinação da normalidade dos dados foi realizada a partir do teste de Shapiro-Wilk e da verificação dos histogramas, adotando-se uma análise paramétrica dos dados. As variáveis contínuas foram expressas por média ± desvio padrão e as variáveis categóricas por número ou percentual. Para verificação da correlação entre o TUG e o VO_2_pico, foi realizado o teste de correlação de Pearson.

Na criação do modelo preditor, foi realizada análise de correlação de Pearson, verificando quais variáveis se relacionavam com o VO_2_pico. Foram analisados: TUG, idade, sexo, índice de massa corporal (IMC), presença de DAC e ou IC, frequência cardíaca, fração de ejeção, pressão arterial sistólica e circunferência da cintura. Atendendo a todos os pressupostos, foram realizadas regressões lineares múltiplas com as variáveis admitidas por significância estatística ou plausibilidade biológica e a construção do modelo preditor com base no TUG foi controlado para: idade, sexo, IMC, circunferência da cintura e pressão arterial sistólica, no intuito de identificar os preditores para o VO_2_pico. O método *stepwise-backward* foi determinado como critério de inclusão e exclusão das variáveis.

Para criação do modelo preditor, foram utilizados dados de 2/3 da amostra total, que compuseram o grupo 1 (criação), admitidos após os critérios de elegibilidade, correspondendo aos 134 primeiros participantes da lista; compondo o grupo 2 (validação), foram empregados 1/3 da amostra total, referente aos 67 participantes restantes da lista. Para comparar a média entre o VO_2_pico determinado (TCPE) e o estimado (modelo preditor) no grupo validação, foi realizado o teste t de Student pareado. A avaliação da concordância entre os métodos foi realizada a partir da análise de Bland-Altman.

O melhor ponto de corte para prever um VO_2_pico ≥ 20 mL.kg^1^.min^1^ foi determinado através da análise da curva ROC, considerando o equilíbrio entre sensibilidade e especificidade no ponto mais próximo de 1 da área abaixo da curva. Para as análises, foi utilizado o software Statistical Package for Social Sciences (SPSS), versão 26.0. Um p < 0,05 foi adotado como limite de significância estatística.

## Resultados

A amostra total (n = 201) foi composta por participantes com idade entre 36 e 92 anos, sendo 72% do sexo masculino. Dentre os participantes, 30% (n = 58) tinham IC e 70% (n = 143) DAC; destes, 58% (n = 81) eram revascularizados. Houve uma predominância de participantes da classe funcional NYHA I na amostra total (53%). No grupo de participantes com DAC, essa classe funcional foi de 60% (n = 69) e com IC foi de 35% (n = 17). Na amostra total, a média de tempo para realização do TUG foi de 7 ± 2,5 segundos e a média do VO_2_pico obtido no TCPE foi de 17 ± 6 mL.kg^1^.min^1^. Quando estratificados por sexo, o desempenho no TUG para os homens foi de 6,86 ± 0,20 segundos e o desempenho para as mulheres foi de 7,23 ± 0,33 segundos. A média do VO_2_pico obtido no TCPE verificado para os homens foi de 18,25 ± 0,50 mL.kg^1^.min^1^ e para as mulheres foi de 15,22 ± 0,57 mL.kg^1^.min^1^ (Tabela 1). A distribuição dos participantes da amostra total e dos grupos criação e validação pode ser visualizada na Tabela 2.

**Tabela 1:** – Dados demográficos, antropométricos, hemodinâmicos, clínicos, farmacológicos, cardiorrespiratórios e funcionais da amostra total, de criação e validação, apresentados por média e desvio padrão, frequência relativa e absoluta n (%)

Variáveis	Total 100% (N=201)	Criação (N=134)	Validação (N= 67)	p
Homens	72% (145)	72% (97)	72% (48)	0,91
Mulheres	28% (56)	28% (37)	28% (19)	0,82
Idade (anos)	67±13	69±13	62±13	0,01*
Peso (kg)	78±16	78±17	77±15	0,59
Altura (cm)	168±9	168±9	169±9	0,01*
IMC (kg/m^2^)	28±5	28±6	27±5	0,91
FE (%)	56%±16	55±17	57±15	0,31
FC (bpm)	69±10	69±10	70±10	0,54
PAS (mmHg)	122±18	122±19	121±16	0,74
PAD (mmHg)	70±10	69±9	73±11	0,01*
SpO_2_	96±2	95±2	95±3	0,29
HAS (%)	60% (120)	63% (84)	54% (36)	0,15
Diabetes (%)	29% (58)	34% (46)	18% (12)	0,01*
Dislipidemia (%)	73% (146)	77% (104)	63% (42)	0,02*
Tabagismo (%)	2,5% (5)	2% (3)	3% (2)	0,75
Valvulopatias (%)	13% (26)	10% (13)	19% (13)	0,06
Cirurgia (%)	42% (84)	44% (59)	37% (25)	0,36
Betabloqueador(%)	78% (152)	78% (104)	76% (48)	0,72
IECA-BRA (%)	69% (139)	76% (100)	62% (39)	0,18
Estatinas (%)	85% (167)	49% (65)	83% (52)	0,47
NYHA I (%)	53% (83)	52% (58)	56% (25)	0,42
II (%)	37% (58)	37% (41)	37% (17)	0,44
III (%)	9% (14)	10% (11)	7% (3)	0,33
IV (%)	0,5% (1)	1% (1)	0%	0,48
TUG (segundos)	7±2,5	7±2,5	6±2	0,01*
VO_2_pico (mL.kg^-1^.min^-1^)	17±6	17±6	18±6	0,18

Comparação das variáveis contínuas: teste T de Student; comparação das variáveis categóricas: teste qui-quadrado de Pearson; * indica significância estatística quando p < 0,05 na análise entre os grupos criação e validação. BRA: bloqueador de receptores da angiotensina; FC: frequência cardíaca; FE: fração de ejeção; HAS: hipertensão arterial sistêmica; IECA: inibidor da enzima conversora da angiotensina; IMC: índice de massa corporal; NYHA: New York Heart Association; PAD: pressão arterial diastólica; PAS: pressão arterial sistólica; SpO_2_: saturação periférica de oxigênio; TUG: timed up and go; VO_2_pico: consumo de oxigênio no pico do esforço.

**Tabela 2:** – Distribuição da amostra por sexo e por faixa etária

Faixa etária	Amostra total		Amostra criação		Amostra validação	

	Homens	Mulheres	Homens	Mulheres	Homens	Mulheres
30 – 39	1	4	0	2	1	2
40 – 49	13	6	6	3	7	3
50 – 59	23	9	14	4	9	5
60 – 69	38	11	21	6	17	5
70 – 79	40	18	33	14	7	4
80 – 89	28	7	21	7	7	0
90+	2	1	2	1	0	0

### Grupo criação

A amostra do grupo criação foi composta por 134 participantes, com média de idade de 69 ± 13 anos, sendo 72% do sexo masculino. A classe funcional dos pacientes identificou neste grupo que 52% eram pertencentes à classe NYHA l e 37% à classe NYHA ll. O desempenho no TUG foi de 7 ± 2,5 segundos e a média do VO_2_pico obtido no TCPE foi de 17 ± 6 mL.kg^1^.min^1^ (Tabela 1).

### Grupo validação

No grupo validação, a amostra foi composta por 67 participantes, com média de idade de 62 ± 13 anos, sendo 72% do sexo masculino. A classe funcional dos pacientes identificou neste grupo que 56% eram pertencentes à classe NYHA l e 37% à classe NYHA ll. O desempenho no TUG foi de 6 ± 2 segundos e a média do VO_2_pico obtido no TCPE foi de 18 ± 6 mL.kg^1^.min^1^ (Tabela 1).

### Criação do modelo preditor

**Figura 1:**
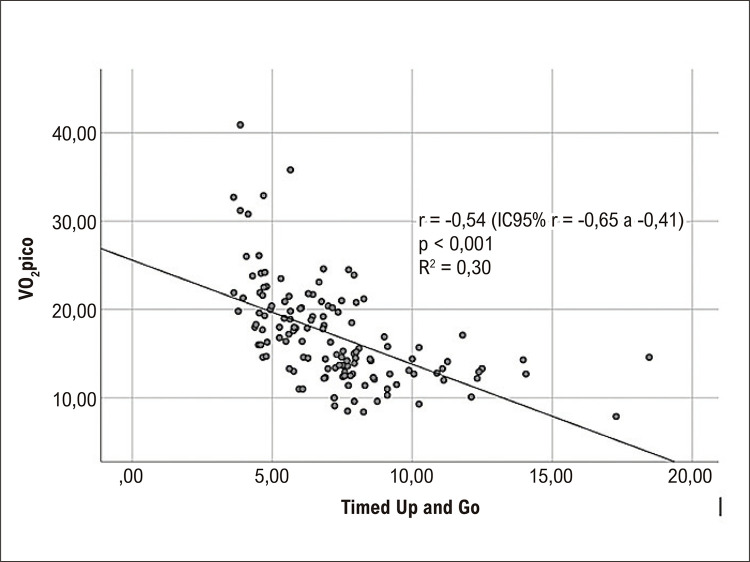
– Análise de correlação de Pearson entre o teste desempenho no TUG e o VO2pico no grupo criação (n = 134). IC: intervalo de confiança; VO2pico: consumo de oxigênio no pico do esforço.

A correlação realizada no grupo criação (n = 134) para verificar a relação entre o TUG e o VO_2_pico identificou um coeficiente de correlação de r = −0,54 (intervalo de confiança de 95%: −0,65 a −0,41; p < 0,001) e um R^2^ de 0,30 (Figura 1).

Foi realizada a regressão linear múltipla com dados do grupo criação (n = 134) para identificar os preditores independentes e desenvolver o modelo para estimar o VO_2_pico com base no TUG. A equação preditiva construída foi: ${VO}_{2}\text{ pico }=33,553+(-0,149*\text{ idade })+(-0,738*\text{ TUG })+(-2,870*\text{ sexo })$ ; sendo atribuído o valor 0 ao sexo masculino e 1 ao sexo feminino (Tabela 3). No modelo final foi encontrado um r de 0,643 e o R^2^ ajustado de 0,400, conforme descrito na Tabela 3.

### Validação da equação preditora

Na equação preditora desenvolvida, foram incluídos os dados da amostra do grupo de validação (n = 67) e foi encontrada uma média de VO_2_pico estimado de 18,81 mL.kg^1^.min^1^. A média de VO_2_pico determinado pelo TCPE nesta amostra foi de 18,18 mL.kg^1^.min^1^ e, após realizar uma análise com o teste t pareado, para comparar as médias entre o VO_2_pico estimado pela equação e o VO_2_pico determinado pelo TCPE, não foi encontrada diferença estatisticamente significativa entre os métodos.

### Análise de concordância

**Figura 2:**
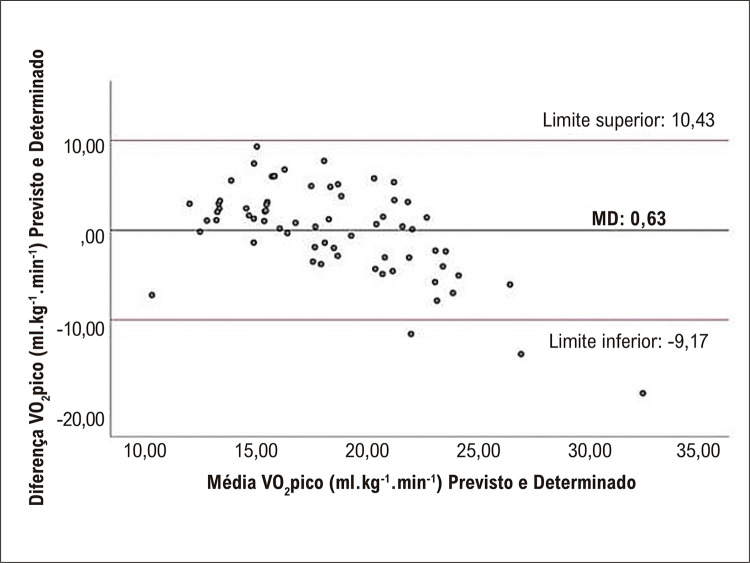
– Análise de concordância através do gráfico de Bland-Altman entre o VO2pico determinado pelo teste cardiopulmonar de exercício e o VO2pico estimado pela equação preditiva. MD: diferença média; VO2pico: consumo de oxigênio no pico do esforço.

A análise do gráfico de Bland-Altman demonstrou que apenas 3 (4,4%) pacientes da amostra de validação (n = 67) estavam fora dos limites superior e inferior de concordância. Estes 3 pacientes eram do sexo masculino, sendo um com 68 anos de idade, acometido por IC e com IMC de 24 kg/m^2^; um segundo com 65 anos de idade, acometido por DAC e com IMC de 25 kg/m^2^e o terceiro com 44 anos, acometido por DAC e com IMC de 24 kg/m^2^, ressaltando ainda que os 3 possuíam dislipidemia. Não foi verificada a presença de viés de proporção nessas análises (Figura 2).

### Determinação de melhor ponto de corte

**Figura 3:**
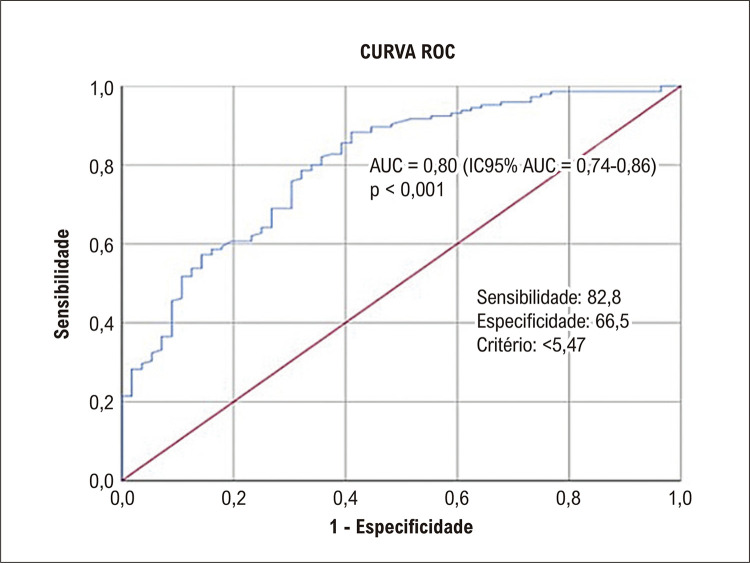
– Curva ROC mostra a capacidade do teste TUG em estimar o VO2pico em pacientes cardiopatas a partir da avaliação da AUC. AUC: área sob a curva; IC: intervalo de confiança; ROC: característica de operação do receptor; TUG: timed up and go; VO2pico: consumo de oxigênio no pico do esforço.

A análise de curva ROC foi realizada com a amostra total (n = 201) e verificou uma área sob a curva de 0,80 (intervalo de confiança de 95%: 0,74 a 0,86), para prever um VO_2_pico ≥ 20 mL.kg^1^.min^1^. O ponto de corte no TUG para prever um VO_2_pico ≥ 20 mL.kg^1^.min^1^ foi de 5,47 segundos, com sensibilidade de 82,8% e especificidade de 66,5% (Figura 3).

## Discussão

Os dados encontrados neste estudo apontam que o TUG apresentou boa associação com o VO_2_pico dos cardiopatas participantes de um programa de reabilitação cardíaca. Foi identificado um ponto de corte para o TUG capaz de identificar cardiopatas com melhor ACR e ele também demonstrou através das análises da equação preditora ser um teste com adequada capacidade preditora na avaliação da ACR nesta população.

O VO_2_pico obtido através da equação preditora elaborada neste estudo com base no desempenho no TUG demonstrou concordância com o VO_2_pico determinado pelo TCPE na mesma amostra, demonstrando ser um método adequado para estimar a ACR de cardiopatas. Em uma metanálise com adultos saudáveis, Kodama et al.^12^sugeriram que a ACR seria um importante preditor de mortalidade e eventos cardiovasculares. Apesar da sua amostra apresentar diferentes características do nosso estudo, é possível inferir que uma melhor ACR está associada a menores riscos de complicações cardiovasculares. É importante frisar que na literatura ainda são escassos os estudos que relacionem o teste TUG à população de pacientes cardiopatas.

A ACR estabelecida pelo VO_2_pico é um importante componente de avaliação da saúde, pois, de acordo com Carvalho et al.^3^ e Ritt et al.,^5^ trata-se de um determinante que deve ser mensurado periodicamente em pacientes cardiopatas, com o intuito de monitorar a CF diante da realização de atividades da vida diária e instrumental. O TCPE pode nem sempre estar acessível à população em geral, principalmente em locais com restrição de recursos materiais, estruturais e de profissionais capacitados. Alternativas com protocolos indiretos validados, com menor complexidade operacional, maior celeridade e menor custo,^3^ como o modelo preditor desenvolvido neste estudo, podem promover uma avaliação da ACR mais abrangente, sendo, portanto, de grande relevância na prática clínica.

Em um estudo com idosos no pré-operatório por diversas naturezas, Boereboom et al.^13^afirmaram que o TUG poderia ser um teste útil para substituir o TCPE quando este não estiver disponível. Entretanto, entendemos que se deve adotar cautela ao sugerir que unicamente o desempenho no teste TUG seja suficiente em substituir o TCPE para avaliar a ACR, principalmente em pacientes cardiopatas. A equação preditora desenvolvida neste estudo propõe uma estimativa mais criteriosa da ACR em cardiopatas do que apenas o tempo de realização no teste TUG, por empregar maior rigor estatístico, além de considerar características da individualidade biológica dos pacientes, portanto, representando um método seguro.

Foi encontrado neste estudo uma correlação moderada negativa entre o desempenho no TUG e o VO_2_pico, fato semelhante aos achados de Pedrosa et al.,^14^ que em um estudo com idosas hipertensas, também encontraram uma correlação moderada negativa entre o TUG e o TC6M, que é um teste funcional correspondente ao TCPE. É importante ressaltar que apesar dos testes diferirem, ambos objetivam mensurar a ACR e ainda que as amostras também tenham características diferentes, a amostra com cardiopatas em nosso estudo identificou que 60% dos participantes também eram portadores de hipertensão arterial sistêmica.

Já no estudo de Lourenço et al.,^15^ foi verificada uma correlação moderada negativa mais expressiva entre o TUG e o TC6M em uma amostra com mulheres adultas portadoras de artrite reumatoide. Todavia, Boereboom et al.,^13^ no seu estudo com idosos, encontraram uma correlação negativa fraca, apesar de significativa, entre o TUG e o TCPE. Estes estudos apresentam divergências nas características sociodemográficas, clínicas, bem como nos seus protocolos de realização dos testes, no entanto, indicam a existência de relação entre os métodos, o que nos permite deduzir que o TUG pode ser um teste com sugestiva capacidade de designar níveis de ACR.

Após as análises, foram considerados preditores do VO_2_pico nesta investigação: a idade, o sexo e o tempo de realização no teste TUG. No que se refere à diferenciação da ACR pelo sexo, o estudo de Herdy et al.^4^ apontou que mulheres saudáveis na mesma faixa etária dos homens apresentavam valores de VO_2_máx que variavam entre 76% a 83% dos valores médios apresentados pelos homens. Já no estudo de Nunes et al.,^16^ foi encontrada uma variação do VO_2_máx por sexo ainda maior, sendo que o sexo feminino apresentou valores médios de VO_2_máx próximos a 70% dos valores atribuídos ao sexo masculino. Os dados dos estudos de Herdy et al.^4^ e de Nunes et al.^16^ assemelham-se aos encontrados neste estudo com cardiopatas, uma vez verificado que o sexo feminino apresentou um valor médio de VO_2_pico de 83% do valor médio atribuído ao sexo masculino, condições que podem ser explicadas pelas diferenças fisiológicas e morfológicas inerentes a cada sexo.

Um outro preditor do VO_2_pico encontrado neste estudo foi a idade, que apresentou uma relação diretamente proporcional ao tempo de realização do TUG e inversamente proporcional ao VO_2_pico obtido no TCPE. Em nossa amostra, foi verificado um desempenho médio no TUG de 7 ± 2,5 segundos, aproximando-se dos valores normativos de 8 ± 1 segundos sugeridos por Bohannon^17^ para pessoas da mesma faixa etária, que ainda apontou a redução gradativa do desempenho TUG a cada aumento de década na idade.

Outros estudos como os de Khant et al.^18^e Bischoff et al.,^19^ apontaram a idade como um fator determinante para o desempenho no TUG, sugerindo que a utilização do TUG na prática clínica não deveria desprezar características biológicas como a idade e o sexo para determinar o desempenho no teste. Deste modo, é importante destacar que quando o objetivo da utilização do TUG for prever a ACR, principalmente de cardiopatas, um modelo preditivo com a utilização do desempenho no TUG, além da utilização de características como a idade e o sexo dos avaliados, bem como o uso das respectivas constantes propostas pelo modelo estatístico, podem garantir uma estimativa mais assertiva.

Nas análises da curva ROC, foi verificado que o TUG demonstrou um nível de acurácia plausível para estimar a ACR em cardiopatas. O ponto de corte encontrado para prever um VO_2_pico ≥ 20 mL.kg^1^.min^1^, ou seja, de indivíduos com melhor ACR, foi de 5,47 segundos, sugerindo como em outros estudos que o TUG pode ser um teste confiável para estimar a ACR em cardiopatas.^4,13^ Em análise com indivíduos com características clínicas diversas no pré-operatório e idade semelhante à da nossa amostra, foi identificado um ponto de corte no TUG de 6,5 segundos para prever comprometimentos no pós-operatório, com base num VO_2_pico < 18,6 mL.kg^1^.min^1^.^13^

Os estudos trazem metodologias diferentes, entretanto, os resultados indicam parâmetros aproximados de desempenho no TUG com amostras de faixa etária equivalente no intuito de prever a ACR. Em outras análises com idosos saudáveis, foram apontados parâmetros diversos para o desempenho no TUG.^17-23^ Deste modo, é importante reconhecer que a determinação de pontos de corte no TUG deveria considerar características clínicas dos avaliados (idade, sexo, peso e ainda comorbidades, altura ou comprimento dos membros inferiores), garantindo maior homogeneidade das amostras e propondo pontos de corte mais precisos.

O baixo desempenho no TUG pode estar relacionado à CF reduzida em idosos cardiopatas e essa relação já havia sido apontada no estudo de Bateman et al.^24^ Além disso, Boereboom et al.^13^apontaram que a performance reduzida no teste estava atrelada ao aumento da incidência de doenças cardiovasculares e à mortalidade, sendo que esses fatores podem estar associados a um processo inflamatório e complicador cardiometabólico derivado do processo de sarcopenia.

Ao construirmos e validarmos uma equação preditiva para estimar o VO_2_pico, propomos uma ferramenta simplificada, capaz de compor o rol de instrumentos para avaliação da CF, contribuindo para uma prática clínica mais completa e abrangente aos portadores de DAC e IC. Os resultados deste estudo podem beneficiar principalmente pacientes que são usuários do Sistema Único de Saúde, uma vez que há uma importante limitação de equipamentos, espaços apropriados, recursos financeiros e equipe profissional disponível para realização de outros testes para a mesma finalidade.

Este estudo apresentou algumas limitações, como o fato de se tratar de um estudo unicêntrico, uma vez que estudos multicêntricos permitem a participação de uma amostra mais representativa de uma população com grande diversidade como a população brasileira. Importante pontuar que o modelo preditor desenvolvido e o ponto de corte identificado no TUG foi proposto para uma amostra de pacientes cardiopatas com DAC e/ou IC. Entendemos que as análises poderiam ser mais específicas, caso portadores de DAC e IC fossem analisados separadamente, conscientes de que estudos com tamanho amostral maior são necessários para este mister. Nosso estudo não trouxe correlação de prognóstico, haja vista tratar-se de um estudo transversal e utilizou um desfecho substituto classicamente relacionado a prognóstico que foi o VO_2_pico.

## Conclusão

O desempenho no TUG associou-se de forma negativa, moderada e significativa com a ACR em uma população de pacientes cardiopatas. Para prever o VO_2_pico com base no desempenho no TUG, foi desenvolvida uma equação e validada apresentando bom desempenho. Um tempo ≤ 5,47 segundos foi o ponto de corte determinado para prever um VO_2_pico ≥ 20 mL.kg^1^.min^1^. Estes resultados podem ajudar na formulação de diretrizes de avaliação da CF nesta população.

**Tabela 3:** – Dados do modelo final com base no TUG obtido na regressão linear múltipla para prever o VO2pico

Variável	Beta	IC95%	p
TUG	–0,738	–1,088 a –0,389	<0,001
Idade (anos)	–0,149	–0,222 a –0,077	<0,001
Sexo feminino	–0,2870	–4,553 a –1,186	<0,001
Constante	33,553	29,274 a 37,831	<0,001

Ajustado para idade, sexo, índice de massa corporal, doença arterial coronariana, insuficiência cardíaca, frequência cardíaca, fração de ejeção, pressão arterial sistólica e circunferência da cintura; significância estatística quando p < 0,05. IC: intervalo de confiança; TUG: timed up and go; VO_2_pico: consumo de oxigênio no pico do esforço.
