# Supplementary material for: Predição do Consumo de Oxigênio de Pico em Pacientes Cardiopatas com Base no Desempenho no Teste Timed Up and Go
Source: Arq Bras Cardiol. 2023 Dec 15;120(12):e20230338. [Article in Portuguese] doi: 10.36660/abc.20230338 (PMC10789370; doi:10.36660/abc.20230338)
Supplement: Translation

# Translation: Prediction of Peak Oxygen Consumption in Patients with Heart Disease Based on Performance on the Timed Up and Go Test

**Authors:** Danilo Silva dos Santos, Ciro Oliveira Queiroz, Cristiane Maria Carvalho Costa Dias, Gerson Cipriano, Queila Oliveira Borges, Luiz Eduardo Fonteles Ritt

**Affiliations:** 1 Brazil Escola Bahiana de Medicina e Saúde Pública – Unidade Acadêmica Brotas, Salvador, BA – Brazil; 2 Brazil Universidade Estadual do Sudoeste da Bahia – Campus de Jequie, Jequie – Brazil; 3 Brazil Universidade de Brasília, Brasília, DF – Brazil; 4 Brazil Hospital Cardio Pulmonar – Centro de Estudos Clínicos, Salvador, BA – Brazil

**Keywords:** Oxygen Consumption, Cardiovascular Diseases, Exercise Test

## Abstract

**Background:**

The use of the timed up and go (TUG) test to assess cardiorespiratory fitness in patients with heart disease has not been well defined in the literature.

**Objectives:**

Test the association between TUG and peak oxygen consumption (VO_2_peak), construct an equation based on TUG to predict VO_2_peak, and determine a cutoff point to estimate VO_2_peak ≥ 20 mL/kg/min.

**Methods:**

This cross-sectional study included 201 patients with coronary artery disease or heart failure, between 36 and 92 years of age, who underwent TUG and cardiopulmonary exercise test. Correlation, ROC curve, multiple linear regression, and Bland-Altman analyses were performed. The significance level was set at p < 0.05.

**Results:**

The mean age of the total sample was 67 ± 13 years, and 70% of participants were male. The mean VO_2_peak was 17 ± 6 mL/kg/min, and the mean TUG time was 7 ± 2.5 seconds. The correlation between VO_2_peak and TUG was r = −0.54 (p < 0.001), and R^2^ was 0.30. The following equation was developed based on $\text{ TUG: }{VO}_{2}\text{ peak }=33.553+(-0.149\times \text{ age })+(-0.738\times \text{ TUG })+(-2.870\times \text{ sex })$ ; a value of 0 was assigned to the male sex and 1 to the female sex (adjusted R: 0.41; adjusted R^2^: 0.40). The VO_2_peak estimated by the equation was 18.81 ± 3.2 mL/kg/min, and the VO_2_peak determined by cardiopulmonary exercise test was 18.18 ± 5.9 mL/kg/min (p > 0.05). The best cutoff point in the TUG for VO_2_peak ≥ 20 mL/kg/min was ≤ 5.47 seconds (area under the curve: 0.80; 95% confidence interval: 0.74 to 0.86).

**Conclusions:**

TUG and VO_2_peak showed a significant association. A prediction equation for VO_2_peak was developed and validated internally with good performance. The cutoff point in the TUG to predict VO_2_peak ≥ 20 mL/kg/min was ≤ 5.47 seconds.

## Introduction

Cardiovascular diseases are the main cause of death worldwide, and they were responsible for 17.9 million deaths in 2019, accounting for 32% of all deaths.^1^ Cardiovascular diseases are highly disabling, leading to decreased functional capacity, a condition that may imply severe cardiovascular risks and that indicates worse prognosis for patients.^2-5^

Functional capacity is the ability to perform daily activities independently, and it is considered an important health indicator, as it is associated with quality of life.^6^ Functional capacity can be assessed by oxygen consumption at peak effort (VO_2_peak), which is the determinant of cardiorespiratory fitness (CRF) for the general population, and VO_2_peak values ≥ 20 mL/kg/min are related to a better prognosis for those evaluated. The cardiopulmonary exercise test (CPET) is the gold-standard method for measuring CRF; however, it is not a very accessible test, as it requires expensive equipment and appropriate facilities, and it needs to be conducted by a specialist, making it a cumbersome and restrictive procedure for the majority of the population.^4,7^

Currently, validated submaximal tests, such as the 6-minute walk test (6MWT) and the 6-minute step test, are viable alternatives to the CPET for assessment of CRF.^5^ It is recommended to periodically measure functional capacity in the population with heart disease, as it is an indicator of clinical functional prognosis and, consequently, mortality.^5,7^ Therefore, if it is impossible to perform CPET or additional functional tests, other instruments capable of functional assessment are used.

The timed up and go (TUG) test assesses functional mobility based on lower limb muscle strength, balance, and agility.^8-10^ It is a simple test, and performance considers the time in seconds for the patient being assessed to stand up from a chair and, as quickly as possible, walk in a straight line for 3 meters, turn around, and return to the chair, sitting down again.^11^ Data on the use and performance of TUG in patients with heart disease are still scarce.

Therefore, the main objective of this study was to construct a prediction equation for VO_2_peak based on TUG time of patients with heart disease, as well as to analyze the association between TUG and VO_2_peak and determine a cutoff point in the TUG to define patients with better CRF.

## Methods

This is a cross-sectional study based on analysis of data from participants in a cardiac rehabilitation program, during the period from August 2017 to March 2020. Following clinical guidelines, patients underwent CPET and TUG, in a reference hospital in cardiology, in the city of Salvador, Bahia, Brazil.

This study included patients affected by coronary artery disease (CAD) and/or heart failure (HF), as diagnosed by the patients’ clinical history (acute myocardial infarction, stable CAD, angioplasty or revascularization procedures, or even presence of angina or dyspnea) and presence of electrocardiographic or echocardiographic abnormalities, using the Simpson method to measure the ejection fraction. Participants who did not undergo CPET and TUG were excluded.

During the initial assessment, clinical and sociodemographic data were collected, and CPET was performed. The CPET was performed using a treadmill (Micromed brand, Centurion 300 model, São Paulo, Brazil), with a gas analyzer (Cortex Inc brand, Metalizer 3b model, Leipzig, Germany), with the capacity to measure each breath. Each patient’s functional class determined the ramp protocol applied, with the goal of standardizing tests that lasted between 8 and 12 minutes. The ventilatory data obtained were analyzed at 10-second intervals and VO_2_peak was expressed in mL/kg/min. To verify perceived exertion, the modified Borg scale was used.

The TUG was performed under the supervision of a trained health professional, within an interval of 2 to 7 days after CPET. For the TUG, a chair was used with a seat 46 cm above the ground, with a backrest and no arm support. In the initial position, the patients being assessed were seated in the chair, leaning back, with their feet flat on the floor. To perform the TUG, participants were instructed that, upon the command “get up and go,” when the stopwatch was started, they should stand up without the help of their arms, walk as quickly as possible and, crossing a line positioned 3 meters from the chair, turn around and return to the chair, sitting down again, at which point the timer was stopped. Performance on the TUG test corresponded to the time in seconds needed to carry out this process, determined by the stopwatch that was administered by an evaluator trained for the protocol.

The study protocol was submitted to the Celso Figueirôa Research Ethics Committee at the Santa Izabel Hospital, and it received approval under CAAE number 57813016.0.3001.5533. The study was conducted in accordance with the Declaration of Helsinki for clinical research and resolution 466/12 of the Brazilian National Council of Health. All study participants signed a free and informed consent form.

### Statistical analysis

Data normality was determined using the Shapiro-Wilk test and by checking the histograms, adopting a parametric analysis of the data. Continuous variables were expressed as mean ± standard deviation and categorical variables as number or percentage. To verify the correlation between TUG and VO_2_peak, the Pearson correlation test was performed.

In creating the prediction model, Pearson correlation analysis was performed, checking which variables were related to VO_2_peak. The following were analyzed: TUG, age, sex, body mass index (BMI), presence of CAD and/or HF, heart rate, ejection fraction, systolic blood pressure, and waist circumference. Taking all assumptions into account, multiple linear regressions were carried out with the variables admitted for statistical significance or biological plausibility, and the construction of the prediction model based on the TUG was controlled for the following: age, sex, BMI, waist circumference, and systolic blood pressure, with the aim of identifying predictors of VO_2_peak. The stepwise backward method was determined as a criterion for inclusion and exclusion of variables.

To create the prediction model, we used data from 2/3 of the total sample admitted after the eligibility criteria, forming group 1 (creation), corresponding to the first 134 participants on the list; group 2 (validation) comprised 1/3 of the total sample, referring to the remaining 67 participants on the list. To compare the mean between the determined VO_2_peak (CPET) and the estimated VO_2_peak (prediction model) in the validation group, paired Student’s t test was used. Bland-Altman analysis was used to assess agreement between methods.

The best cutoff point to predict a VO_2_peak ≥ 20 mL/kg/min was determined by means of ROC curve analysis, considering the equilibrium between sensitivity and specificity at the point closest to 1 of the area under the curve. For the analyses, Statistical Package for Social Sciences (SPSS) software, version 26.0, was used. The limit of statistical significance was set at p < 0.05.

## Results

The total sample (n = 201) included participants between 36 and 92 years of age, and 72% of them were male. Among the participants, 30% (n = 58) had HF, and 70% (n = 143) CAD; 58% (n = 81) of these patients were revascularized. There was a predominance of participants in NYHA functional class I in the total sample (53%). In the group of participants with CAD, 60% were in this functional class (n = 69), as were 35% of participants with HF (n = 17). In the total sample, the mean TUG time was 7 ± 2.5 seconds, and the mean VO_2_peak obtained in the CPET was 17 ± 6 mL/kg/min. When stratified by sex, TUG time was 6.86 ± 0.20 seconds in men and 7.23 ± 0.33 seconds in women. The mean VO_2_peak obtained in the CPET was 18.25 ± 0.50 mL/kg/min for men and 15.22 ± 0.57 mL/kg/min for women (Table 1). The distribution of participants in the total sample and the creation and validation groups is displayed in Table 2.

**Table 1:** – Demographic, anthropometric, hemodynamic, clinical, pharmacological, cardiorespiratory, and functional data of the total sample, creation sample, and validation sample, presented as mean and standard deviation or relative and absolute frequency n (%)

Variables	Total 100% (N=201)	Creation (N=134)	Validation (N= 67)	p
Men	72% (145)	72% (97)	72% (48)	0.91
Women	28% (56)	28% (37)	28% (19)	0.82
Age (years)	67±13	69±13	62±13	0.01*
Weight (kg)	78±16	78±17	77±15	0.59
Height (cm)	168±9	168±9	169±9	0.01*
BMI (kg/m^2^)	28±5	28±6	27±5	0.91
EF (%)	56%±16	55±17	57±15	0.31
HR (bpm)	69±10	69±10	70±10	0.54
SBP (mmHg)	122±18	122±19	121±16	0.74
DBP (mmHg)	70±10	69±9	73±11	0.01*
SpO_2_	96±2	95±2	95±3	0.29
SAH (%)	60% (120)	63% (84)	54% (36)	0.15
Diabetes (%)	29% (58)	34% (46)	18% (12)	0.01*
Dyslipidemia (%)	73% (146)	77% (104)	63% (42)	0.02*
Tobacco use (%)	2.5% (5)	2% (3)	3% (2)	0.75
VHD (%)	13% (26)	10% (13)	19% (13)	0.06
Surgery (%)	42% (84)	44% (59)	37% (25)	0.36
Beta blocker (%)	78% (152)	78% (104)	76% (48)	0.72
ACEI/ARB (%)	69% (139)	76% (100)	62% (39)	0.18
Statins (%)	85% (167)	49% (65)	83% (52)	0.47
NYHA I (%)	53% (83)	52% (58)	56% (25)	0.42
II (%)	37% (58)	37% (41)	37% (17)	0.44
III (%)	9% (14)	10% (11)	7% (3)	0.33
IV (%)	0.5% (1)	1% (1)	0%	0.48
TUG (seconds)	7±2.5	7±2.5	6±2	0.01*
VO_2_peak (mL.kg^-1^.min^-1^)	17±6	17±6	18±6	0.18

Comparison of continuous variables: Student’s t test; comparison of categorical variables: Pearson’s chi-square test; * indicates statistical significance with p < 0.05 in the analysis between the creation and validation groups. ACEI: angiotensin-converting enzyme inhibitor; ARB: angiotensin receptor blocker; BMI: body mass index; DBP: diastolic blood pressure; EF: ejection fraction; HR: heart rate; NYHA: New York Heart Association; SAH: systemic arterial hypertension; SBP: systolic blood pressure; SpO_2_: peripheral oxygen saturation; TUG: timed up and go test; VHD: valvular heart diseases; VO_2_peak: peak oxygen consumption.

**Table 2:** – Distribution of the samples by sex and age range

Age range	Total sample		Creation sample		Validation sample	

	Men	Women	Men	Women	Men	Women
30 to 39	1	4	0	2	1	2
40 to 49	13	6	6	3	7	3
50 to 59	23	9	14	4	9	5
60 to 69	38	11	21	6	17	5
70 to 79	40	18	33	14	7	4
80 to 89	28	7	21	7	7	0
90+	2	1	2	1	0	0

### Creation group

The sample of the creation group consisted of 134 participants, with a mean age of 69 ± 13 years, and 72% of them were male. In this group, 52% of patients were in NYHA functional class l, and 37% were in NYHA ll. Performance on the TUG was 7 ± 2.5 seconds, and the mean VO_2_peak obtained in the CPET was 17 ± 6 mL/kg/min (Table 1).

### Validation group

In the validation group, the sample consisted of 67 participants, with a mean age of 62 ± 13 years, and 72% of them were male. In this group, 56% of patients were in NYHA functional class l, and 37% were in NYHA ll. Performance on the TUG was 6 ± 2 seconds, and the mean VO_2_peak obtained in the CPET was 18 ± 6 mL/kg/min (Table 1).

### Creation of the prediction model

The analysis carried out in the creation group (n = 134) to verify the relationship between TUG and VO_2_peak identified a correlation coefficient of r = −0.54 (95% confidence interval: −0.65 to −0.41; p < 0.001) and an R^2^ of 0.30 (Figure 1).

**Figure 1:** – Pearson correlation analysis between performance on the TUG and VO2peak in the creation group (n = 134). CI: confidence interval; VO2peak: peak oxygen consumption.

Multiple linear regression was performed with data from the creation group (n = 134) to identify independent predictors and develop the model to estimate VO_2_peak based on TUG. The following prediction equation was constructed: ${VO}_{\text{, peak }}=33.553+(-0.149\times \text{ age })+(-0.738\times TUG)+(-2.870\times \mathrm{sex})$ ; a value of 0 was assigned to the male sex and 1 to the female sex (Table 3). The final model verified an r of 0.643 and an adjusted R^2^ of 0.400, as displayed in Table 3.

**Table 3:** – Data from the final model based on the TUG obtained in multiple linear regression to predict VO**2**peak

Variable	Beta	95% CI	p
TUG	–0.738	–1.088 to –0.389	<0.001
Age (years)	–0.149	–0.222 to –0.077	<0.001
Female sex	–0.2870	–4.553 to –1.186	<0.001
Constant	33.553	29.274 to 37.831	<0.001

Adjusted for age, sex, body mass index, coronary artery disease, heart failure, heart rate, ejection fraction, systolic blood pressure, and waist circumference; statistical significance when p < 0.05. CI: confidence interval; TUG: timed up and go; VO_2_peak: peak oxygen consumption.

### Validation of the prediction equation

In the prediction equation developed, data from the sample of the validation group (n = 67) were included, and an estimated mean VO_2_peak of 18.81 mL/kg/min was found. The mean VO_2_peak determined by the CPET in this sample was 18.18 mL/kg/min, and, after conducting an analysis with the paired t test to compare the means between the VO_2_peak estimated by the equation and the VO_2_peak determined by the CPET, no statistically significant difference was found between the methods.

### Agreement analysis

Analysis of the Bland-Altman plot demonstrated that only 3 (4.4%) patients in the validation sample (n = 67) were outside the upper and lower limits of agreement. These 3 patients were male. One was 68 years old, with HF and BMI of 24 kg/m^2^; the second was 65 years old, with CAD and BMI of 25 kg/m^2^, and the third was 44 years old, with CAD and BMI of 24 kg/m^2^. It is worth underscoring that all 3 had dyslipidemia. The presence of proportion bias was not verified in these analyses (Figure 2).

**Figure 2:** – Bland-Altman plot for agreement analysis between the VO2peak determined by the cardiopulmonary exercise test and the VO2peak estimated by the prediction equation. MD: mean difference; VO2peak: peak oxygen consumption.

### Determination of best cutoff point

ROC curve analysis was performed with the total sample (n = 201), and it verified an area under the curve of 0.80 (95% confidence interval: 0.74 to 0.86), to predict VO_2_peak ≥ 20 mL/kg/min. The TUG cutoff point to predict VO_2_peak ≥ 20 mL/kg/min was 5.47 seconds, with sensitivity of 82.8% and specificity of 66.5% (Figure 3).

**Figure 3:** – ROC curve showing the capacity of the TUG to estimate VO2peak in patients with heart disease based on AUC evaluation. AUC: area under the curve; CI: confidence interval; ROC: receiver operating characteristic; TUG: timed up and go test; VO2peak: peak oxygen consumption.

## Discussion

The data found in this study indicate that TUG showed a good association with VO_2_peak in patients with heart disease participating in a cardiac rehabilitation program. We identified a cutoff point in TUG capable of identifying heart disease patients with better CRF, and analysis of the prediction equation also demonstrated that it is a test with adequate predictive capacity in the assessment of CRF in this population.

The VO_2_peak obtained by the prediction equation developed in this study based on TUG performance demonstrated agreement with the VO_2_peak determined by the CPET in the same sample. This demonstrates that it is an appropriate method for estimating the CRF of patients with heart disease. In a meta-analysis with healthy adults, Kodama et al.^12^ suggested that CRF would be an important predictor of mortality and cardiovascular events. Although their sample presents different characteristics from our study, it is possible to infer that better CRF is associated with lower risks of cardiovascular complications. It is important to emphasize that there are still few studies in the literature that relate TUG to the population of patients with heart disease.

CRF established by VO_2_peak is an important component of health assessment; according to Carvalho et al.^3^ and Ritt et al.,^5^ it is a determinant that must be measured periodically in patients with heart disease, with the aim of monitoring functional capacity when carrying out activities of daily living and instrumental activities. CPET may not always be accessible to the general population, especially in places with limitations in material and structural resources and trained professionals. Alternatives with validated indirect protocols, with less operational complexity, greater speed, and lower cost,^3^ such as the prediction model developed in this study, can promote a more comprehensive assessment of CRF and are therefore of great relevance in clinical practice.

In a study with preoperative elderly patients of different natures, Boereboom et al.^13^ stated that TUG could be a useful test to replace CPET when it is not available. Nonetheless, we understand that caution should be adopted when suggesting that performance on the TUG alone is sufficient to replace CPET in the assessment of CRF, especially in patients with heart disease. The prediction equation developed in this study proposes a more careful estimate of CRF in patients with heart disease than just the time taken to perform the TUG test, as it employs greater statistical rigor, in addition to considering patients’ individual biological characteristics, thus representing a safe method.

This study found a moderate negative correlation between TUG performance and VO_2_peak, similar to the findings of Pedrosa et al.,^14^ who, in a study with elderly female patients with hypertension, also found a moderate negative correlation between TUG and 6MWT, which is a functional test corresponding to CPET. It is important to underscore that, although the tests differ, the objective of both is to measure CRF; although the samples also have different characteristics, the sample with heart disease in our study indicated that 60% of the participants also had systemic arterial hypertension.

The study by Lourenço et al.^15^ found a more expressive moderate negative correlation between TUG and 6MWT in a sample of adult women with rheumatoid arthritis. On the other hand, Boereboom et al.,^13^ in their study with elderly patients, found a weak, albeit significant, negative correlation between TUG and CPET. These studies present divergences in sociodemographic and clinical characteristics, as well as in their testing protocols; however, they indicate the existence of a relationship between the methods, which allows us to deduce that TUG may be a test with a suggestive ability to determine CRF levels.

After the analyses, the following were considered predictors of VO_2_peak in this investigation: age, sex, and time to complete the TUG test. Regarding the differentiation of CRF by sex, the study by Herdy et al.^4^ showed that healthy women in the same age group as men had VO_2_max values that ranged between 76% and 83% of the mean values found for men. On the other hand, the study by Nunes et al.,^16^ found an even greater variation in VO_2_max by sex, with women presenting mean VO_2_max values close to 70% of the values found in men. The data from the studies by Herdy et al.^4^ and Nunes et al.^16^ are similar to those found in our study of patients with heart disease, as we found the mean VO_2_peak value of female participants was 83% of the mean value of males. These conditions that can be explained by the physiological and morphological differences inherent to each sex.

Another predictor of VO_2_peak found in this study was age, which presented a directly proportional relationship to time taken to complete the TUG and an inversely proportional relationship to the VO_2_peak obtained in the CPET. In our sample, mean TUG time was 7 ± 2.5 seconds, approaching the normative values of 8 ± 1 seconds suggested for patients in the same age group by Bohannon,^17^ who also indicated a gradual reduction in TUG performance for each decade increase in age.

Other studies, such as those by Nency et al.^18^ and Heike et al.^19^ indicated age as a determining factor for performance on the TUG, suggesting that use of the TUG in clinical practice should not neglect biological characteristics, such as age and sex to determine test performance. Accordingly, it is important to highlight that, when the objective of using the TUG is to predict CRF, especially in patients with heart disease, a prediction model using TUG time, in addition to the use of characteristics such as patients’ age and sex, using the respective constants proposed by the statistical model, can guarantee a more assertive estimate.

The ROC curve analyses verified that the TUG demonstrated a plausible level of accuracy for estimating CRF in patients with heart disease. The cutoff point found to predict VO_2_peak ≥ 20 mL/kg/min, or be it, patients with better CRF, was 5.47 seconds, suggesting, as in other studies, that the TUG can be a reliable test to estimate CRF in patients with heart disease.^4,13^ An analysis of preoperative patients with diverse clinical characteristics and age similar to that of our sample identified a cutoff point of 6.5 seconds on the TUG to predict postoperative complications, based on VO_2_peak < 18.6 mL/kg/min.^13^

Studies have used different methodologies; however, the results indicate approximate parameters for performance on the TUG with samples of equivalent age range, with the aim of predicting CRF. In other analyses with healthy elderly patients, different parameters were identified for performance on the TUG.^17-23^ Accordingly, it is important to recognize that determination of cutoff points in the TUG should consider clinical characteristics of the patients being assessed (age, sex, weight, comorbidities, height, or length of the lower limbs), thus guaranteeing greater sample homogeneity and proposing more precise cutoff points.

Low performance on the TUG may be related to reduced functional capacity in elderly patients with heart disease, and this relationship had already been indicated in the study by Cordeiro et al.^24^ Furthermore, Boereboom et al.^13^ indicated that reduced test performance was linked to increased incidence of cardiovascular diseases and mortality, and these factors may be associated with an inflammatory process and cardiometabolic complications derived from the process of sarcopenia.

By constructing and validating a prediction equation to estimate VO_2_peak, we propose a simplified tool, which could become part of the list of instruments for assessing functional capacity, contributing to more complete and comprehensive clinical practice for patients with CAD and HF. The results of this study can mainly benefit patients who are users of the Brazilian Unified Health System, given that there are important limitations to equipment, appropriate spaces, financial resources, and professional teams available to carry out other tests for the same purpose.

This study has some limitations, for instance, the fact that it was a single-center study, seeing that multicenter studies allow the participation of a more representative sample of a highly diverse population, such as the Brazilian population. It is important to point out that the prediction model developed and the cutoff point identified in the TUG were proposed for a sample of patients with CAD and/or HF. We understand that the analyses could have been more specific if patients with CAD and HF had been analyzed separately, and we are aware that studies with a larger sample size are necessary for this task. Our study did not provide a correlation with prognosis, seeing that it was a cross-sectional study using a surrogate outcome classically related to prognosis, namely, VO_2_peak.

## Conclusion

Performance on the TUG was negatively, moderately, and significantly associated with CRF in a population of patients with heart disease. To predict VO_2_peak based on TUG performance, an equation was developed and validated, showing good performance. Time ≤ 5.47 seconds was the cutoff point determined to predict VO_2_peak ≥ 20 mL/kg/min. These results can assist in formulating guidelines for assessing functional capacity in this population.
